# Innate ability, health, motivation, and social capital as predictors of students’ cognitive, affective and psychomotor learning outcomes in secondary schools

**DOI:** 10.3389/fpsyg.2022.1024017

**Published:** 2022-10-28

**Authors:** Valentine Joseph Owan, John Asuquo Ekpenyong, Onyinye Chuktu, Michael Ekpenyong Asuquo, Joseph Ojishe Ogar, Mercy Valentine Owan, Sylvia Okon

**Affiliations:** ^1^Department of Educational Foundations, University of Calabar, Calabar, Nigeria; ^2^Ultimate Research Network (URN), Calabar, Nigeria; ^3^Department of Educational Management, University of Calabar, Calabar, Nigeria; ^4^Institute of Education, University of Calabar, Calabar, Nigeria; ^5^Department of Psychology, Caritas University, Amorji-Nike, Enugu State, Nigeria

**Keywords:** health, innate ability, motivation, social capital, factor analysis, structural equation modeling

## Abstract

**Background:**

Previous studies assessing students’ learning outcomes and identifying contributing factors have often dwelt on the cognitive domain. Furthermore, school evaluation decisions are often made using scores from cognitive-based tests to rank students. This practice often skews evaluation results, given that education aims to improve the three learning domains. This study addresses this gap by assessing the contributions of four students’ input to their cognitive, affective and psychomotor skills (CAPs).

**Methods:**

A cross-section of senior secondary class II students (*n* = 870), sampled through the multistage procedure, participated in a physical survey. Students’ Inputs Questionnaire (STIQ) and Learning Outcomes Questionnaire (LOQ) were used for data collection. Based on data obtained from a pilot sample (*n* = 412), principal axis factoring (PAF) was performed to assess the internal structure of the instruments following an oblique rotation. The KMO value of sampling adequacy were 0.88 and 0.94, while the Bartlett’s test of sphericity were significant χ^2^(253) = 5,010; *p* < 0.001 and χ^2^(105) = 3693.38, *p* < 0.001 for the STIQ and LOQ, respectively. Confirmatory factor analysis was used to assess the models’ acceptability based on the maximum likelihood estimation technique. The main study used hierarchical linear regression for data analysis.

**Results:**

Findings indicated that innate ability, health, motivation and social capital relatively and cumulatively predicted students’ overall, cognitive, affective and psychomotor learning outcomes. The proportion of variance explained by the predictors increased at different levels of the models with the addition of new variables. Students’ social capital reduced the effect of students’ innate ability regardless of their motivation and health status.

**Conclusion/implication:**

This study has provided evidence that the four students’ inputs are crucial predictors of their learning outcomes in the three domains. This result is helpful for school management to provide services aimed at improving the school climate for students’ motivation and social capital. The result can provide policymakers with a proper understanding of the constituents of learning outcomes and how policies can be aligned to secure quality student inputs for maximum productivity in education.

## Introduction

The primary reason for teaching and learning is the modification and repositioning of students’ cognitive, affective and psychomotor skills at all levels of education. Research has shown that an adequate evaluation of students’ learning in schools must focus on these three crucial aspects of learning (Sönmez, 2017; Bitok, 2020; Noor et al., 2020). The need for holistic evaluation of students’ learning is prompted by the fact that students’ success is central to the analysis of the education process (Afkhaminia et al., 2018; Abushandi, 2021). Studies have also established that students’ learning achievements are critical parameters for assessing whether or not education goals have been maximized (Ali, 2013; Etor et al., 2019). Therefore, assessment of students’ achievements across the three domains of learning is worthwhile in holistically addressing issues associated with the teaching and learning process in schools and is also apt in evaluating the extent to which the school system’s goals are achieved. It is established that when students perform beyond the average standard set by society in skills acquisition, cognitive performance and affective attributes, they are considered excellent and as resources that can contribute meaningfully to society’s future development (Olaitan, 2017).

Due to the importance of the subject, much research focus in the last two decades has investigated different factors with the central aim of improving students’ learning outcomes (e.g., Casey and Goodyear, 2015; Meshulam et al., 2021; Baabdullah et al., 2022; Villena-Taranilla et al., 2022). For instance, a study has proven that students’ breakfast and snack consumption is positively associated with academic performance, cognitive function and physical activity (Masoomi et al., 2020). Similarly, the effect of learners’ demographic characteristics on academic performance has also been extensively considered by researchers globally (Thoren et al., 2016; Owan et al., 2019; Owan, 2020; Aduma et al., 2022). Such characteristics differ across populations and may be attributed to students’ genetic traits, experience, and choice (Boogert et al., 2018; Reitan and Stenberg, 2019; Mõttus et al., 2020).

Specifically, studies have shown that students are educational inputs in the education production process, and their characteristics can affect the quality of educational outcomes attainable in any school system (Apple et al., 2016; Espinosa, 2017). These characteristics range from gender, age, innate ability, health status, motivation and social relations (Hindal et al., 2013a). Therefore, it is crucial to understand how these variables affect students’ learning outcomes in the cognitive, affective and psychomotor domains. In fact, while many studies have addressed students’ poor learning outcomes, the majority have only focused on the cognitive domain. The cognitive domain focuses on the students’ mental, intellectual and thinking abilities; the affective domain focus on learners’ emotional and sociological abilities; whereas the psychomotor domain deals with students’ physical, skill-based or kinaesthetic abilities. According to Hoque (2016), the cognitive domain concerns students’ knowledge, whereas the affective and psychomotor domains concern students’ attitudes and skills. Since these domains focus on different learner traits, they should be measured independently in relation to other predictors. Consequently, the current study was developed to predict students learning outcomes across the three domains based on their innate ability, health, motivation and social capital.

## Literature review

### Studies on innate ability

Innate ability is a natural talent or skill that has been with a living thing from its inception. It is not a learnt habit but an internal feature of the organism. For example, intelligence can be an inborn trait that students possess from their genetic makeup/composition. It is a known fact in cognitive psychology that genetic (just like environmental) factors are essential for students’ cognitive development and ability (Hill et al., 2018; Coleman et al., 2019; Deary et al., 2022). The literature has well documented that students’ innate ability is crucial for all subjects and determines the degree of school success (Leslie et al., 2015; Meyer et al., 2015; Ito and McPherson, 2018; Heydera et al., 2020). It has been found that students with good abilities achieve better (Cornelisz and van Klaveren, 2018; Yu, 2020; O'Connell and Marks, 2021; Melawati et al., 2022), especially in subjects such as mathematics (Semeraro et al., 2020), and algebra (O'Connell et al., 2018). Furthermore, a study which used a machine learning algorithm discovered that students with low cognitive abilities face the risks of having mental breaks during classes (Srimaharaj et al., 2018), and such mental breaks affect their learning achievements (Owan et al., 2022).

However, the cited studies all measured students’ achievement from the cognitive domain, with some studies being experimental in nature. Thus, the documented relationship between students’ ability and achievement is well-known in the cognitive domain. Nevertheless, the degree to which students’ ability predict their learning outcomes in the affective and psychomotor domains remains unclear. To show the importance of the two aspects often ignored in the literature, Espinosa (2017) used cross-sectional empirical data from Vietnam to illustrate the education process of cognitive and emotional (affective) abilities. Affective abilities were analyzed as an educational outcome to quantify the production of educational results in a more realistic way. Results showed that children’s inherent qualities impacted their cognitive and affective outcomes. Despite focusing on the two domains, Espinosa’s study did not consider the psychomotor aspect of learning. Consequently, the current study was undertaken to plug these knowledge gaps in the literature by linking students’ innate ability to learning outcomes across the three domains (cognitive, affective and psychomotor).

### Studies on health

Students’ health refers to the state of physical, social, and psychological wellbeing among learners in a given school. “It is related to the promotion of wellbeing, the prevention of mental disorders, and the treatment and rehabilitation of people affected by mental disorders” (Obumse & Egenti, 2021, p. 119). A study has documented that school failure, grade repetition, and dropout are more likely for ill pupils than those who are otherwise healthy (Shaw et al., 2015), indicating that the health status of students is a determinant factor of their success in schools. According to McIsaac et al. (2015), nutritional deficiencies in the brain and body impact a child’s dietary condition. Another student input affecting their learning outcomes across the three learning domains is intrinsic motivation (Sivrikaya, 2019). Accordingly, those who excelled across several tasks were more divergent and visual–spatial in their thinking, had better working memory, and were less reliant on a specific study area (Hindal et al., 2013b). With this in mind, researchers found that students’ attitudes and reasoning skills significantly predicted their academic performance (Vilia et al., 2017; Luo et al., 2021; Greisel et al., 2022).

Similarly, McIsaac et al. (2015) revealed that students with low academic performance were likelier to have unhealthy habits than good ones. They further revealed that food quality, physical activity, sugary beverage intake, breakfast skipping and not participating in physical exercise during the morning break were all statistically significant associations. The cited research found that encouraging students to engage in healthy behaviors might boost their academic performance. The findings cited above support that students’ health is a significant predictor of educational outcomes, though the study only measured the cognitive dimension of educational outcomes.

### Studies on motivation

Motivation is the degree to which an individual chooses to direct his or her attention and efforts toward a goal (Brown, 2007). To be “motivated” implies providing the impetus for oneself to make an effort toward a specific objective. A favorable association between student motivation and gains in reading comprehension (Sari and Suprapti, 2017) and achievement (Whitney and Bergin, 2018) has been shown. However, Marsela (2017) found that student enthusiasm to learn had a modest impact on students’ ability to read and comprehend. Studies have documented that students’ motivation relates positively to their academic achievement (Momany et al., 2015) and learning outcomes (Georgiou and Kyza, 2018; Won et al., 2020; Cho et al., 2021; Howard et al., 2021). More specifically, it was discovered that students’ self-test scores for extrinsic motivation rise when their academic success rises and vice versa (Sivrikaya, 2019; Wagbara and Furo, 2021).

To prove the strength of the relationship, there have been numerous studies showing a modest correlation between student motivation and achievement (Rosmayanti and Yanuarti, 2018), especially in science (Khin and Swe, 2018) and English language (Ndruru et al., 2022). However, other researchers found a weak positive correlation between motivation and students’ academic achievement in English (Dewangga and Nasaruddin, 2020; Rahardjo and Pertiwi, 2020). The disagreement in the results of the cited studies is attributable to various factors such as geographic differences where they were conducted, demographic differences of the respondents used, or the methodology and design used. Variations in the results of the literature reviewed may also be due to the motivational profiles of respondents. For instance, a study performed a latent profile analysis and found that students in Ohio showed differences in cognitive and social engagement and academic performance based on their motivational profiles (Xie et al., 2020). The result of Xie and his colleagues suggest that students with different levels and types of motivation achieve differently in school.

A Meta-analysis of empirical studies published between 2011 and 2019 was conducted by Boudadi and Gutiérrez-Colón (2020). Two key conclusions were reached from the assessment of 68 publications culled from the top scholarly databases (Scopus and Web of Science), of which only 15 were included in the analysis. Results found many studies on motivation and engagement, but only a few demonstrated obvious correlations with learning outcomes. According to the findings, there seem to be no robust associations between gamification, motivation, and cognitive operations. Thus, the researchers found a gap and recommended more studies on the three variables to bridge the lacuna. On that note, the current study was undertaken to further expand the scope of the literature on motivation by focusing on Nigerian secondary school students.

### Studies on social capital

The concept of social capital is a powerful tool for analyzing why some students are more successful than others in the school. Students’ achievement relies heavily on parental social capital, which includes their hopes, duties, and the extensive social networks they have built for their children at home, in the classroom, and the wider society. The cultural norms and values that encourage students’ efforts, the school’s disciplinary and academic atmosphere, and parents’ expectations and responsibilities for their children’s education all play a role in the wide range of student achievement. In order to explain social patterns and processes that lead to racial gaps in school success, Coleman (1988) established the notion of social capital. He said that parental participation and financial commitment to a child’s education are significant determinants of academic performance and that these factors are also influenced by the educational expectations, norms, and duties within a family or society. There is a correlation between countries’ social capital levels and their citizens’ academic achievement (Mishra, 2020). However, high levels of social capital have been linked in some studies to a decrease in opportunities for personal and societal development. More research is required to determine the drawbacks of a strong social capital (Paxton, 1999; Woolcock and Narayan, 2000).

**Figure 1:**
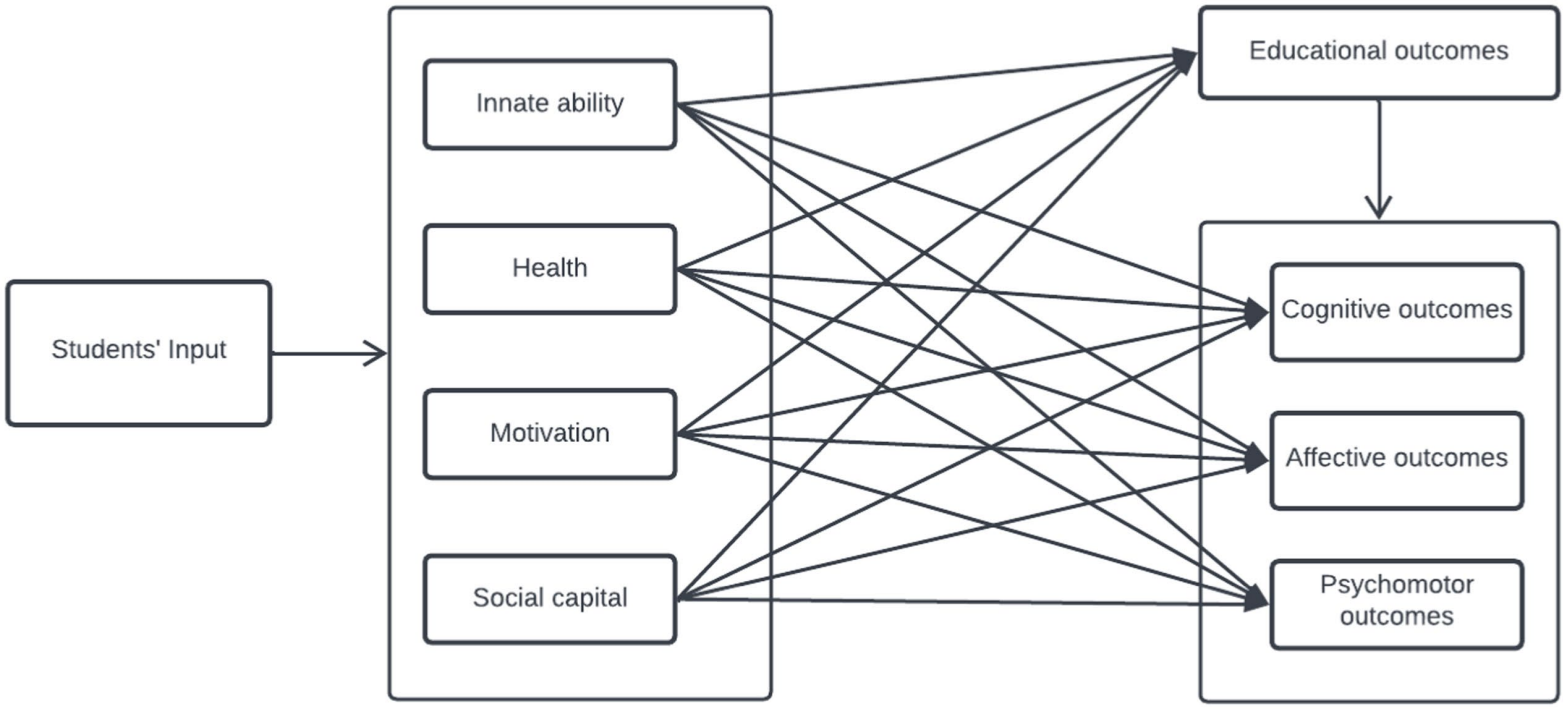
Conceptual model of the study showing the predictive and criterion variables.

Similarly, other researchers have identified social relationships as a determinant of students’ learning outcomes (Cho et al., 2007; Gablinske, 2014). A child’s connection with classmates also substantially impacts educational results (Espinosa, 2017). Nevertheless, learning outcomes in the cited studies were assessed mainly from the cognitive domain. In fact, the review of Kuranchie and Addo (2017) revealed various research gaps in the social capital theory and difficulties that educators need to address to fully realize the concept’s potential. The researchers found that their social capital favorably impacted students’ educational results. They noted that the cognitive component of learning outcomes has received more attention and recommended that future studies be more inclusive by focusing on the affective and psychomotor dimensions of learning outcomes. The present study addresses this gap raised by the review of Kuranchie and Addo by linking four students’ inputs (students’ innate ability, health, motivation and social capital) to the three areas of learning outcomes – cognitive, affective and psychomotor skills (CAPs). Therefore, this study’s conceptual model is presented in Figure 1.

Figure 1 shows four students’ inputs: innate ability, health, motivation and social capital. Furthermore, the central dependent variable is educational outcomes with three dimensions: cognitive, affective, and psychomotor. We hypothesized that the four students’ input directly predicts their learning outcomes generally and in the three dimensions. In Figure 1, all predictive relationships are direct, without any indirect or mediation effect. Hierarchical regression analysis was performed in the current study to analyze the contributions of these variables individually and collectively to students’ learning outcomes generally and across the three dimensions.

## Materials and methods

### Research design and participants

The research design adopted for this study was the cross-sectional research design. The cross-sectional design allows for direct observation of the phenomena investigated and analysis of the information collected at one time to produce faster results. The population of this study comprised 53,255 students (males = 26,206; females = 27,047) spread across 87 public secondary schools in Calabar Education Zone, Nigeria.

**Figure 2:**
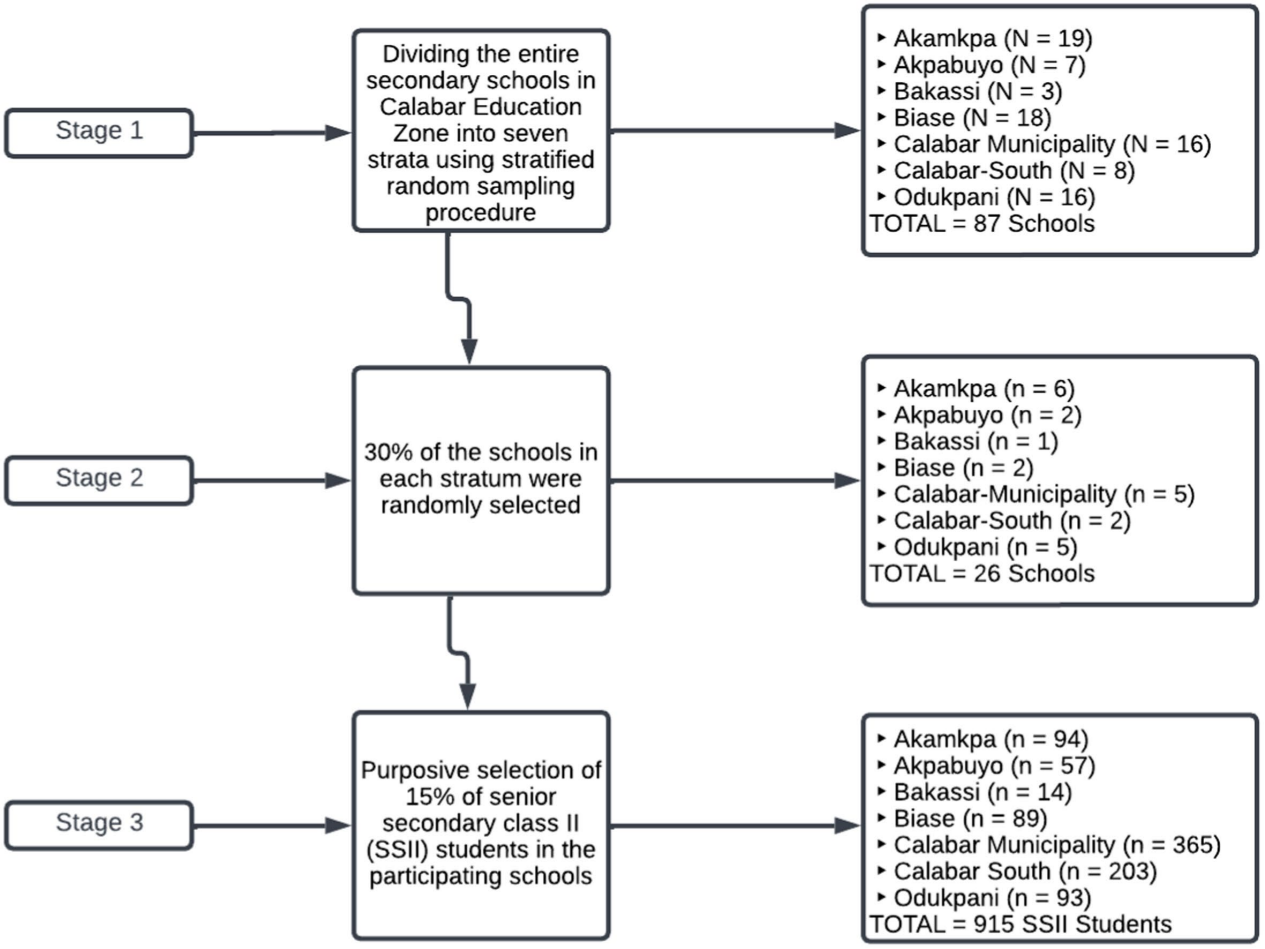
The multistage sampling procedure of this study.

The Multistage sampling procedure involving stratified proportionate and purposive sampling techniques was used for this study. At stage 1, stratified random sampling was used to divide the entire secondary schools in Calabar Education Zone into seven strata according to Local Education Authorities (LEAs) available (such as Akamkpa, Akpabuyo, Bakassi, Biase, Calabar-Municipality, Calabar-South and Odukpani). In stage 2, 30% of the schools in each LEA (stratum) were randomly selected, resulting in an overall selection of 26 schools for the study. Similarly, in stage 3, we purposively selected 15% of senior secondary class II (SSII) students in the participating schools as the sample for this study. Thus, a sample of 915 SSII students was randomly selected for the study. This sample is distributed according to the LEAs as follows: Akamkpa (*n* = 94), Akpabuyo (*n* = 57), Bakassi (*n* = 14), Biase (*n* = 89), Calabar Municipality (*n* = 365), Calabar South (*n* = 203), and Odukpani (*n* = 93). The sampling procedure of this study is pictorially depicted in the flowchart in Figure 2. The respondents’ biodata reveals that 44.1% (*n* = 384) are males while 55.9% (*n* = 486) are females. For students’ age, 48.9% (*n* = 425) are between 10 and 20 years, while 51.1% (*n* = 445) are 21 years or older. Furthermore, 31.7% of the respondents (*n* = 276) are of schools in rural locations, 33.8% (*n* = 294) attend schools in semi-urban areas and 34.5% (*n* = 300) are students of urban schools. Over half of the students (*n* = 439, 50.5%) are from families with high socioeconomic status, whereas 49.5% (*n* = 431) are from families with low socioeconomic status. Similarly, 48.2% (*n* = 419) of the students are from families with an intact structure, while 51.8% (*n* = 451) are from broken families.

### Instrumentation

Two instruments served the purpose of data collection in this study – the students’ inputs Questionnaire (STIQ) and Learning Outcomes Questionnaire. The researchers designed both instruments due to an absence of an existing instrument in the study’s setting. STIQ comprised 26 items, whereas LOQ contained 30 items. The items in both instruments were generated based on an extensive review of related literature and previous instruments. Students’ input is defined as the characteristics of students (such as their gender, age, innate ability, health status, motivation, and social relations) that can affect how they learn in schools. A sample item from the STIQ is that *I always struggle hard before understanding what teachers teach in the classroom.* Learning outcome is operationally defined as the extent to which there is a relatively permanent and observable change in students’ cognitive, affective and psychomotor attributes (CAPs) due to their interaction with the (school) environment. A sample item from the LOQ is *I can carry out laboratory experiments independently.* LOQ was designed to measure two dimensions of learning outcomes - students’ affective and psychomotor. Students’ cognitive skill (the third aspect of learning outcome) was measured directly using average sessional results scores across all the subjects. Students’ average scores for all the subjects in the three terms were recorded for the selected students as their scores for cognitive skills.

### Validity and reliability of the instruments

Quantitative content validity of the research instruments was carried out using 10 independent experts (four psychometrists and six Educational Managers). These experts comprised five males and five females, all professors with over 10 years of academic experience. The six educational managers were from specific fields such as economics of education (*n* = 2), policy analysis (*n* = 2), and educational administration and planning (*n* = 2). Two psychometrists had double doctorate degrees in educational psychology and psychological testing, whereas the other two were PhD holders of educational measurement and evaluation. The experiences and diversity of these individuals qualified them as experts suitable for assessing the items in the instruments. The experts independently judged the items’ degree of relevance and clarity to their measured domains.

All the items were scored for relevance and clarity, with higher scores representing higher relevance and clarity of items to their measured domains. The results indicated that the item-level Content Validity Index (I-CVI) realized for STIQ ranged from 0.83–0.99 (for relevance) and 0.91–0.99 (for clarity), while the Scale Content Validity Indices (S-CVI) was 0.98 for both relevance and clarity. For ELOQ, I-CVIs ranged from 0.90–0.99 (for relevance) and 0.84–0.99 for (clarity), whereas S-CVI was 0.99 and 0.98 for relevance and clarity, respectively. The recommendations for revising, dropping and retaining an item as given by different researchers are as follows: For two experts, CVI must be at least 0.80; for three to five experts should be 0.99; for six experts, at least 0.83; six to eight experts, at least 0.83; 9 to 10 experts, at least 0.78 (Polit et al., 2007; Yusoff, 2019). Given that all Item-level Content Validity Indices (I-CVIs) and the Scale-level Content Validity Indices (S-CVIs) for all items and scales of the research instrument are within the range of 0.80 to 0.99, respectively, all the items were retained with minor corrections made per the suggestion of the experts.

We conducted a pilot study to determine the dimensionality and factorial structure of the instruments used for data collection. SSII students (*n* = 412) were randomly selected from non-participating schools in the population to take part in the pilot study. The data collected were subjected to an initial screening using box plots (to check for outliers and normality) and inter-item correlation (for non-clustered items). These were done following the recommendations of instrument validation studies (e.g., Field, 2005; Bassey et al., 2020; Owan et al., 2021). The preliminary screening test did not identify any non-clustered items across all the instruments since different items correlated with other items in the matrix. An exploratory factor analysis (EFA) using principal axis factoring (PAF) was performed to understand the dimensionality and factorial structure of the two instruments, with the extraction based on Eigenvalues greater than 1. The default iteration time was maintained using the promax rotation to suppress factor loadings less than 0.30. The analysis was aided using SPSS (version 26), statistical software. Confirmatory factor analysis (CFA) was used to examine the goodness of fit of the models, while Cronbach alpha reliability was used as a measure of internal consistency (more on EFA, CFA and reliability in the result section).

### Ethical consideration

A comprehensive validity and regulatory data-gathering procedure helped minimize any possible bias in the research. Since completing a survey posed no significant danger to the participants, ethical approval was waived under the Nigerian Code for Health Research Ethics (NCHRE), which exempts survey research (see https://bit.ly/3pK9ORh for further information on this). Written informed consent was received from the respondents since all the participants signed a form confirming that they were aware of the research and were willing to participate. For anonymity, respondents were told that the data gathered would be de-identified and anonymised according to safe harbor standards. The survey participants were informed that their replies would be anonymised before being compiled for objectivity and privacy. A range of equal intervals was used for all biodata (age, gender, family type, parent socioeconomic status) to ensure that no one could be identified from aggregated data. Students’ names were only collected to enable us to get an average score of their sessional results, and after recording them, their names will not be entered during the coding process. The coded data was stored in a computer accessible only to the researchers, with a security system (strong password, antivirus software, and a firewall) to prevent unauthorized access to the obtained data. All participants were notified that their responses would be analyzed aggregately and published in a journal.

### Data collection and analysis

The instruments were administered physically to the respondents at the selected schools with the help of seven trained research assistants. Permission to administer was granted by the principals and vice-principals of the schools following a previous letter of intent. Only students who accepted voluntarily participated in the study. After 2 months of the exercise, we retrieved 870 completed copies of the 915 questionnaires administered. There was an attrition rate of approximately 5%. A preliminary assessment of the returned copies of the instruments revealed no missing data since respondents were given 3 days to fill and return their copies. All responses were scored with a schedule prepared to guide the coding process. Hierarchical regression analysis was used for data analysis with SPSS (version 26) software.

### Model specification

In fitting the hierarchical regression models, the general form of the simple and multiple linear regression equation was used, such as:


(1) Ỳ=βX+ε

While the standardized multiple linear regression equation is given as:


(2) $$Ỳ=\beta_{1}X_{1}+\beta_{2}X_{2}+\beta_{3}X_{3}+\ldots \beta nXn$$

Where: Ỳ = The dependent variable to be predicted.

β_,_ β_1_–β_n_ = The standardized regression coefficients of the predictor variables.

X_1_–X_n_ = The predictor variables.

e = the error term in the model.

From the equations (1) and (2), the specific hierarchical regression models of this study were derived as follows:


(3) $$\mathrm{Model}\;1:LO=\beta_{IA}+\varepsilon \;\left(R^{2}\right)$$

(4) $$\mathrm{Model}\;2:LO=\beta_{IA}+\beta_{SH}+\varepsilon \;\left(R^{2},\;\Delta R^{2}\right)$$

(5) $$\mathrm{Model}\;3:LO=\beta_{IA}+\beta_{SH}+\beta_{SM}+\varepsilon \;\left(R^{2},\;\Delta R^{2}\right)$$

(6) $$\mathrm{Model}\;4:LO=\beta_{IA}+\beta_{SH}+\beta_{SM}+\beta_{SSC}+\varepsilon \;\left(R^{2},\;\Delta R^{2}\right)$$

(7) $$\mathrm{Model}\;1:CS=\beta_{IA}+\varepsilon \;\left(R^{2}\right)$$

(8) $$\mathrm{Model}\;2:CS=\beta_{IA}+\beta_{SH}+\varepsilon \;\left(R^{2},\;\Delta R^{2}\right)$$

(9) $$\mathrm{Model}\;3:CS=\beta_{IA}+\beta_{SH}+\beta_{SM}+\varepsilon \;\left(R^{2},\;\Delta R^{2}\right)$$

(10) $$\mathrm{Model}\;4:CS=\beta_{IA}+\beta_{SH}+\beta_{SM}+\beta_{SSC}+\varepsilon \;\left(R^{2},\;\Delta R^{2}\right)$$

(11) $$\mathrm{Model}\;1:AS=\beta_{IA}+\varepsilon \;\left(R^{2}\right)$$

(12) $$\mathrm{Model}\;2:AS=\beta_{IA}+\beta_{SH}+\varepsilon \;\left(R^{2},\;\Delta R^{2}\right)$$

(13) $$\mathrm{Model}\;3:AS=\beta_{IA}+\beta_{SH}+\beta_{SM}+\varepsilon \;\left(R^{2},\;\Delta R^{2}\right)$$

(14) $$\mathrm{Model}\;4:AS=\beta_{IA}+\beta_{SH}+\beta_{SM}+\beta_{SSC}+\varepsilon \;\left(R^{2},\;\Delta R^{2}\right)$$

(15) $$\mathrm{Model}\;1:PS=\beta_{IA}+\varepsilon \;\left(R^{2}\right)$$

(16) $$\mathrm{Model}\;2:PS=\beta_{IA}+\beta_{SH}+\varepsilon \;\left(R^{2},\;\Delta R^{2}\right)$$

(17) $$\mathrm{Model}\;3:PS=\beta_{IA}+\beta_{SH}+\beta_{SM}+\varepsilon \;\left(R^{2},\;\Delta R^{2}\right)$$

(18) $$\mathrm{Model}\;4:PS=\beta_{IA}+\beta_{SH}+\beta_{SM}+\beta_{SSC}+\varepsilon \;\left(R^{2},\;\Delta R^{2}\right)$$

Notes from equations (3)–(18); LO = Educational outcomes generally; CS = Cognitive skills; AS = Affective skills; PS = Psychomotor skills; IA = Innate ability; SH = Students’ health; SM = Students’ motivation; SSC = Students’ social capital; R^2^ = Coefficient of determination from the regression analysis; ΔR^2^ = The change in the coefficient of determination due to the inclusion of new variables at different levels; ε = the stochastic error term (residual).

## Results

### Exploratory factor analysis

The STIQ was evaluated for dimensionality using the PAF based on the data collected from the 412 students who responded to the instrument in the pilot study. The correlation determinant matrix was used to examine the correlation among the factors—all the items correlated with one or more other items in the matrix. The KMO value of sampling adequacy was 0.88, while the Chi-square associated with Bartlett’s test of sphericity was significant, χ^2^(253) = 5,010; *p* < 0.001. This indicates the suitability of the sample size and data for factor analysis. The PAF analysis revealed a four-factor solution that jointly accounted for 59.54% of the variance explained. A review of the scree plot further indicated that only factors had Eigenvalues greater than 1. The specific loadings of the various items to their respective latent factors ranged from 0.73 to 0.82 (see Table 1). After examining the extracted factors and the items loading to them, the four factors were named students’ social capital (factor 1), students’ health (factor 2), students’ motivation (factor 3), and students’ innate ability (factor 4). Factors 1, 2, 3, and 4 accounted for 17.35%, 15.51%, 14.3%, and 12.37% of the cumulative variance explained.

**Table 1:** Exploratory factor analysis of the structure of the STIQ.

Factors	Items	*M*	*SD*	λ	
				EFA	CFA
Students’ social capital α = 0.89	STI15—I always share ideas with my classmates in school	3.49	1.70	0.82	0.82
	STI18—Having a cordial relationship with fellow students distracts me	3.50	1.69	0.81	0.81
	STI20—I understand better when I study alone	3.51	1.69	0.79	0.79
	STI19—I enjoy group study with other students	3.53	1.68	0.77	0.76
	STI17—My friendship with other students stops at the school	3.55	1.71	0.76	0.77
	STI16—I do not like working with my peers in school	3.58	1.76	0.74	0.75
Students’ health α = 0.89	STI26—I have headaches frequently	3.38	1.69	0.80	0.79
	STI22—I rarely fall sick	3.39	1.71	0.78	0.78
	STI24—I eat meals with plenty of meat every day	3.38	1.69	0.78	0.78
	STI23—Many days, I go to school without food	3.41	1.70	0.77	0.77
	STI25—Sometimes, I sleep in class during lessons	3.43	1.73	0.76	0.76
	STI21—I am always very active in extra-curricular activities	3.43	1.69	0.73	0.72
Students’ motivation α = 0.90	STI11—I have a deep interest in s subject area	3.42	1.71	0.76	0.76
	STI9—Sometimes, I get tired of attending classes	3.47	1.72	0.76	0.76
	STI12—My parents forced me to offer my subject area	3.57	1.70	0.76	0.76
	STI13—I always want to participate in class discussion	3.42	1.73	0.75	0.75
	STI14—Sometimes, I feel like I should stop going to school	3.40	1.71	0.74	0.74
	STI10—I do not like missing classes	3.45	1.73	0.73	0.73
Students’ innate ability α = 0.90	STI7—I always struggle before understanding what is taught	3.48	1.76	0.81	0.81
	STI4—Sometimes, I need additional effort to understand my subjects	3.44	1.72	0.79	0.78
	STI5—I still perform poorly academically despite putting in my best	3.41	1.71	0.78	0.78
	STI6—I do not have difficulties understanding my subjects	3.42	1.736	0.78	0.78
	STI8—I find it very easy to pass my examinations	3.52	1.753	0.76	0.77
Instrument Total	Kaiser-Meyer-Olkin (KMO) = 0.88				
	Bartlett’s Test of Sphericity at 253 df = 5010.42, *p* < 0.05				
	Cronbach Alpha = 0.78				
	Corr. Det. Matrix = 0.000				

Extraction Method: Principal Axis Factoring. Rotation Method: Promax with Kaiser Normalization. e Rotation converged in five iterations.

For the LOQ, an inter-item correlation matrix revealed a determinant value of 0.000, which diverges from the identity matrix with a value of 0.00001. The inter-item correlation analysis revealed three dysfunctional items (LO4, LO9 and LO11). These three items did not correlate with any other item in the matrix, not even among themselves. They were excluded from the Exploratory factor analysis (EFA) because of their loneliness, among other items. The KMO value of sampling adequacy was 0.94, while Bartlett’s test of sphericity was significant, χ^2^(105) = 3693.38, *p* < 0.001. All these indicated that using PAF was plausible, given the data from the sample of 412 students in the pilot study. The analysis yielded a two-factor solution with a cumulative variance of 54.21% explained. The scree plot also revealed two notable factors with Eigenvalues greater than 1. The factors were named based on the nature of the items loaded onto them, as shown in Table 2. Factor loadings ranged from 0.83 to 0.86 for factor 1 (affective skills) and from 0.50 to 0.60 for factor 2 (psychomotor skills). The two factors accounted for 42.35% and 11.86% of the shared variance.

**Table 2:** Exploratory factor analysis of the structure of the LOQ.

Label	Items	*M*	*SD*	λ	
				EFA	CFA
Affective skills α = 0.90	LO10—I have regard for the views of others	3.46	1.70	0.86	0.86
	LO8—The disciplinary measures of my school are uncomfortable	3.48	1.69	0.86	0.86
	LO1—I think good about myself always	3.51	1.66	0.85	0.85
	LO7—I keep school rules and regulations	3.37	1.66	0.84	0.84
	LO3—Sometimes, I doubt my ability to do something well	3.56	1.69	0.83	0.83
	LO2—I always want to lead other people	3.48	1.71	0.83	0.83
	LO6—I like to study independently	3.53	1.72	0.83	0.83
	LO12—I give respect to teachers who deserve it	3.47	1.71	0.83	0.83
	LO5—I am always ready to solve the problem of other students	3.43	1.70	0.83	0.83
Psychomotor skills α = 0.71	EO17—I can carry out laboratory experiments independently	3.45	1.66	0.60	0.60
	EO13—I can operate the computer effectively	3.43	1.74	0.54	0.55
	EO14—I have adequate skills to handle agricultural activities	3.42	1.70	0.54	0.54
	EO18—I have the skills to handle practical exercises	3.31	1.69	0.54	0.55
	EO15—I am very effective in craftwork	3.40	1.72	0.52	0.52
	EO16—I can manipulate mechanized instructional materials	3.27	1.65	0.50	0.50
Instrument total	Kaiser-Meyer-Olkin (KMO) = 0.94				
	Bartlett’s Test of Sphericity at 105 df = 3693.38, *p* < 0.05				
	Cronbach Alpha = 0.83				
	Corr. Det. Matrix = 0.000				

Extraction Method: Principal Axis Factoring. Rotation Method: Promax with Kaiser Normalization. f Rotation converged in three iterations.

### Confirmatory factor analysis

The confirmatory factor analysis (CFA) was performed based on the Maximum Likelihood (ML) estimation technique. Analysis of Moment Structure (Amos v.23) aided the analysis. “The CFA determines how well the variables measured their respective factors (constructs); evaluates the acceptability or otherwise of hypothesized models based on theoretical models; verifies whether the relationships established by the EFA between observed variables (indicators) and their supposed constructs exist” (Owan et al., 2021; p.11). The CFA was applied to all the instruments to determine how various items could measure their targeted latent constructs in this study.

**Figure 3:**
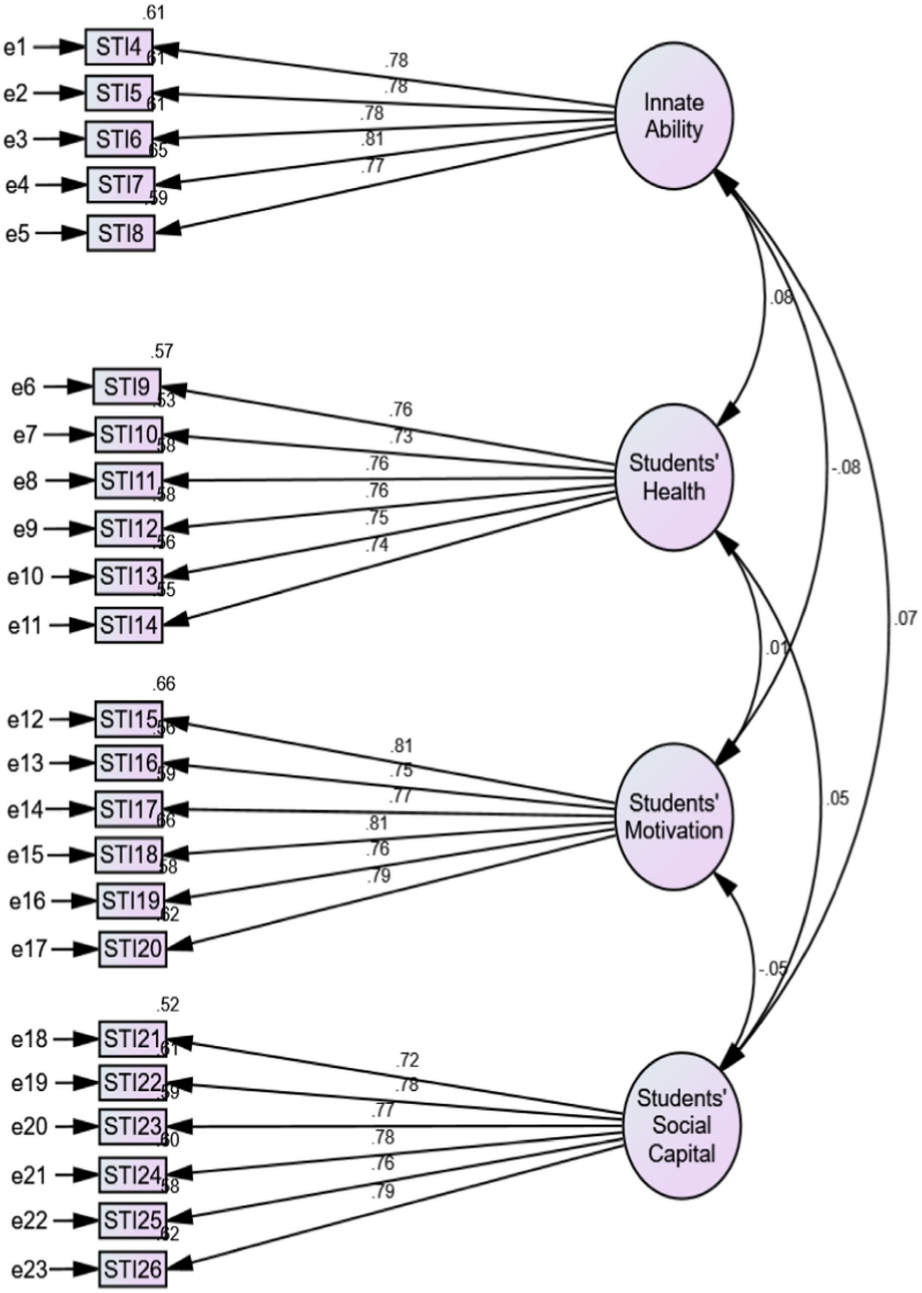
Standardized latent-trait CFA model of the STIQ.

**Figure 4:**
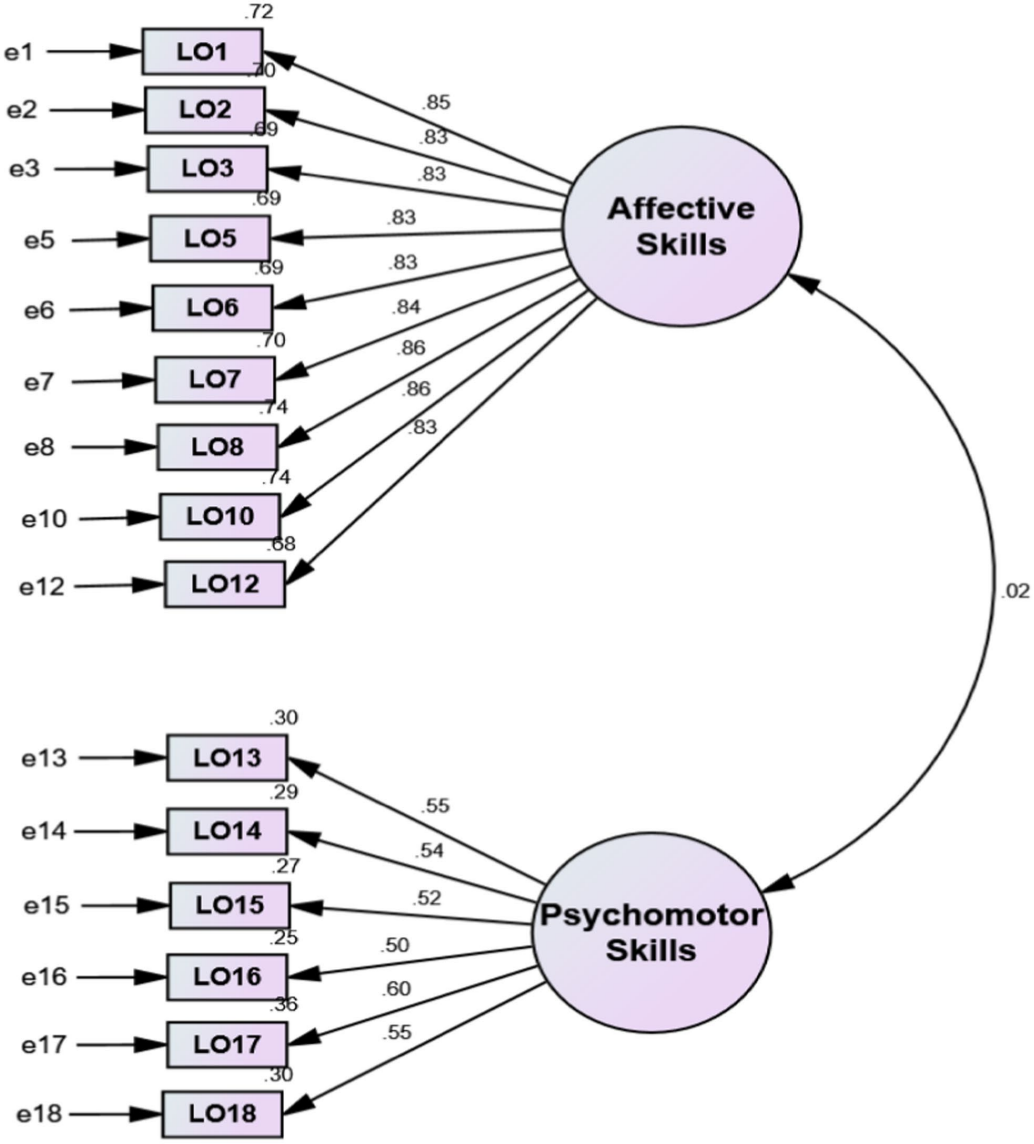
Standardized latent-trait CFA model of the LOQ.

It was further used to verify the authenticity or otherwise of the results of the exploratory factor analysis earlier presented in Tables 1, 2. The CFA results were placed with the EFA in Tables 1, 2 for clarity and comparativeness. However, the CFA models are presented in Figures 3, 4 according to the two instruments. As may be seen in Tables 1, 2 or Figures 3, 4, the CFA loadings of the various items per construct were the same or approximately so, indicating that the results of the EFA were valid and the dimensionality in the various instruments was theoretically sound. Just like the revelation of the inter-item correlation matrix, the result of the CFA also revealed that some items (LO4, LO9 and LO11) were dysfunctional in the models, respectively. Thus, they were removed from the models and eliminated from the questionnaire used for the main study.

The fits of the established CFA models to their existing theoretical models were evaluated using various fit indices. Various fit indices were used due to their overlapping strengths and weaknesses and based on the recommendations of instrument validation studies. For example, Kline (2016) recommended that at least four fit indices (χ^2^, RMSEA, CFI, and SRMR) should be used to decide whether to accept a CFA model. For the CFA models of this study, a broad spectrum of eight carefully selected fit indices was used to determine whether or otherwise to accept them. These include the use of Chi-Square, “Goodness of Fit Index” (GFI), “Normed Fit Index” (NFI), “Relative Fit Index” (RFI), “Comparative Fit Index” (CFI), HOELTER’s Critical N, “Incremental Fit Index” (IFI), “Root Mean Square Error of Approximation” (RMSEA), and “Tucker-Lewis Index” (TLI).

An assessment of the models revealed that the STIQ CFA model had a significant Chi-square value, χ^2^ (224) = 265.89, *p* < 0.05. Going by the Chi-square criteria, the model does not fit the data. However, the Chi-square criterion is widely reported to be sensitive to sample sizes (Gatignon, 2010; Owan et al., 2020), resulting in a Type I error (Myers et al., 2010). Values of 0.95, 0.95, 0.94., 0.99, and 0.99 were obtained for the GFI, NFI, RFI, IFI, TLI, and CFI, respectively. These statistics range from 0.00 to 1.00, with higher values suggesting a better fit (Hooper et al., 2008). However, subsequent tests have shown that a value larger than 0.90 is required to assure the adoption of misspecified models (Hu and Bentler, 1999). All SEM algorithms currently employ these fit indices (TLI and CFI) since they are among the metrics least impacted by sample size (Fan et al., 1999; Shi et al., 2019). A model fit of 0.80 or greater for the Tucker-Lewis Index (TLI) has been proposed as a good model fit (Hooper et al., 2008). However, it has been argued that TLI values of 0.95 suggest a strong model fit (Hu and Bentler, 1999). All the values for the STIQ model were greater than 0.90; hence the model can be said to have an acceptable fit by these criteria. Lastly, an RMSEA value of 0.02 was obtained for the STIQ model. The RMSEA value reflects how well the model fits the population’s covariance matrix when parameter estimates are unknown but optimum (Bassey et al., 2019). The RMSEA score is between 0 and 1, with lower values indicating a better fit to the model (Hu and Bentler, 1999). According to Brown (2015), an RMSEA value of 0.06 or less suggests a satisfactory model fit.

For the LOQ model, the Chi-square criterion yielded a significant value, χ^2^(89) = 167.80, *p* < 0.001. For the GFI, NFI, RFI, IFI, TLI, and CFI, values of 0.95, 0.96, 0.95, 0.98, 0.97, and 0.98 were obtained, respectively. Furthermore, an RMSEA value of 0.05 was obtained for the LOQ model. These are all within acceptable values except for the Chi-Square criterion, perhaps due to the large sample used for the pilot study. Nevertheless, there was sufficient evidence to retain both models. The Cronbach Alpha reliability technique was used to establish the internal consistency of the instruments. The factors’ reliability coefficients (internal consistency) ranged from 0.89 to 0.90 for STIQ and 0.71 to 0.90 for LOQ, respectively (see Tables 1, 2).

### Prediction of students’ inputs on their learning outcomes in terms of CAPs

According to Table 3, the first students’ input (innate ability) contributes 4% to learning outcomes generally in secondary schools. Including students’ health in model 2 increased the contribution of students’ input from 4% to 8%, a 4% increase. In model 3, students’ motivation was added, and the model saw an increase in learning outcomes, generally from 8% to 11%, indicating a 3% increase. In model 4, adding students’ social capital brought about a 2% change in the contribution of students’ input to learning outcomes generally, taking the cumulative contribution of model 4% to 13%. These results determined that students’ innate ability, health, motivation, and social capital contribute to learning outcomes generally by 4%, 4%, 3%, and 2%, respectively. In a composite sense, students’ inputs contribute to learning outcomes generally by 13%. The relative and composite contributions of students’ input were substantial.

**Table 3:** Hierarchical regression results summary of the relative and composite contributions of students’ inputs to learning outcomes generally and in terms of CAPs.

Variables	Model	*R*	*R^2^*	*Adj. R^2^*	*SE*	Δ*R^2^*	Δ*F*	df_1_	df_2_
Learning outcomes generally	1	0.20^a^	0.04	0.04	201.31	0.04	36.04^***^	1	868
	2	0.28^b^	0.08	0.08	197.27	0.04	36.88^***^	1	867
	3	0.33^c^	0.11	0.11	194.01	0.03	30.35^***^	1	866
	4	0.37^d^	0.13	0.13	191.57	0.02	23.19^***^	1	865
Cognitive skills	1	0.20^a^	0.04	0.04	193.16	0.04	34.94^***^	1	868
	2	0.28^b^	0.08	0.08	189.37	0.04	36.03^***^	1	867
	3	0.33^c^	0.11	0.10	186.32	0.03	29.63^***^	1	866
	4	0.36^d^	0.13	0.13	184.04	0.02	22.64^***^	1	865
Affective skills	1	0.26^a^	0.07	0.07	5.25	0.07	65.26^***^	1	868
	2	0.36^b^	0.13	0.12	5.09	0.06	55.57^***^	1	867
	3	0.42^c^	0.17	0.17	4.95	0.04	49.76^***^	1	866
	4	0.45^d^	0.21	0.20	4.86	0.04	35.86^***^	1	865
Psychomotor skills	1	0.26^a^	0.07	0.07	3.09	0.07	62.36^***^	1	868
	2	0.35^b^	0.13	0.12	3.00	0.06	57.68^***^	1	867
	3	0.41^c^	0.17	0.17	2.92	0.04	46.79^***^	1	866
	4	0.45^d^	0.20	0.20	2.87	0.03	34.23^***^	1	865

a Predictors: (Constant), Innate ability.

b Predictors: (Constant), Innate ability, Students’ health.

c Predictors: (Constant), Innate ability, Students’ health, students’ motivation.

d Predictors: (Constant), Innate ability, Students’ health, students’ motivation, Students’ social capital.

*** *p* < 0.001.

In terms of students’ cognitive skills, the first students’ input (innate ability) contributes 4% to learning outcomes in secondary schools in model 1, according to the information in Table 3. The addition of student health to model 2 raised the proportion of variance explained by students’ input from 4% to 8% (an increase of 4%). In model 3, student motivation was included, and the model observed an improvement in learning outcomes from 8% to 11% (suggesting a 3% increment). Including students’ social capital in model 4 increased the variance explained by students’ input to learning outcomes by approximately 2%, bringing the total contribution of model 4%–13%. Based on these findings, it was concluded that students’ innate ability, health, motivation, and social capital all contribute 4%, 4%, 3%, and 2% to learning outcomes regarding cognitive skills, respectively. In a broad sense, students’ input contributes 13% to learning outcomes regarding cognitive skills. The relative and total contributions of student input were found to be significant.

Regarding affective skills, the information in model 1 of Table 3 reveals that the first students’ input (innate ability) contributes 7% to learning outcomes in secondary schools. Including students’ health in model 2 increased the percentage contribution of students’ input from 7% to 13%, indicating a 6% improvement. Model 3 included a third variable (student motivation), and the model showed a 4 per cent increase in learning outcomes in terms of affective skills from 13% to 17%. Adding students’ social capital to model 4 boosted the percentage contribution of students’ input to learning outcomes in affective skills by 4%, bringing model 4’s overall contribution to 21%. These results determined that innate ability, health, motivation, and social capital contribute 7%, 6%, 4%, and 4% to learning outcomes in affective skills. Cumulatively, students’ input accounts for 21% of the total variance in learning outcomes in affective skills, with the remaining 79% of the unexplained variance due to other factors not included in model 4. Students’ input was shown to substantially impact learning outcomes, both relatively and compositely.

Regarding psychomotor skills, model 1 of Table 3 shows that students’ input (innate ability) contributes 7% to secondary school education results at the first level. The addition of students’ health in model 2 raised the percentage contribution of students’ input from 7% to 13%, suggesting a 6% increase. Model 3 included a third variable (student motivation), resulting in a 4% improvement in educational outcomes in psychomotor skills from 13% to 17%. With the inclusion of students’ social capital in Model 4, the percentage contribution of students’ input to learning outcomes in psychomotor skills increased by approximately 3%, bringing model 4’s total contribution to 20%. Based on these findings, it was concluded that innate ability, health, motivation, and social capital all contribute 7%, 6%, 4%, and 3% to learning outcomes in psychomotor skills, respectively. In terms of psychomotor skills, students’ input cumulatively accounts for 20% of the overall variation in learning outcomes, with the remaining 80% of the unexplained variance attributable to other variables not included in model 4. The contribution of students’ input relatively and cumulatively to learning outcomes in terms of psychomotor skills was substantial.

The relative predictions of the specific students’ inputs were used to fit the hierarchical regression models earlier specified for this study (see Table 4). Table 4 shows that students’ health, motivation and social capital made significant unique predictions to learning outcomes generally and in terms of CAPs in public secondary schools. Students’ innate ability made significant unique contributions to learning outcomes in terms of affective and psychomotor skills; however, the unique prediction of students’ innate ability was not significant in the model of their learning outcomes generally and in the aspect of cognitive skills in public secondary schools. This means that students’ social capital weakened students’ innate ability prediction. This suggests that students’ social ties with other colleagues boost their overall performance but diminish how they generally rely on their inherent abilities to achieve academic success. The following hierarchical regression models are fitted based on Table 4.


(19) $$\mathrm{Model}\;1:LO=0.20_{IA}+201.31\;\left(0.04\right)$$

(20) $$\mathrm{Model}\;2:LO=0.17_{IA}+0.20_{SH}+197.27\;\left(0.08,\;0.04\right)$$

(21) $$\begin{array}{l} \mathrm{Model}\;3:LO=0.12_{IA}+0.16_{SH}+0.19_{SM}+\\ 194.01\;\left(0.11,\;0.03\right) \end{array}$$

(22) $$\begin{array}{l} \mathrm{Model}\;4:LO=0.07_{IA}+0.13_{SH}+0.14_{SM}+0.18_{SSC}\\ +191.57\;\left(0.13,\;0.02\right) \end{array}$$

(23) $$\mathrm{Model}\;1:CS=0.20_{IA}+193.16\;\left(0.04\right)$$

(24) $$\mathrm{Model}\;2:CS=0.16_{IA}+0.20_{SH}+189.37\;\left(0.08,\;0.04\right)$$

(25) $$\begin{array}{l} \mathrm{Model}\;3:CS=0.11_{IA}+0.16_{SH}+0.19_{SM}\\ +186.32\;\left(0.11,\;0.03\right) \end{array}$$

(26) $$\begin{array}{l} \mathrm{Model}\;4:CS=0.07_{IA}+0.13_{SH}+0.14_{SM}+0.18_{SSC}\\ +\;184\;\left(0.13,0.02\right) \end{array}$$

(27) $$\mathrm{Model}\;1:AS=0.26_{IA}+5.25\;\left(0.07\right)$$

(28) $$\mathrm{Model}\;2:AS=0.23_{IA}+0.24_{SH}+5.09\;\left(0.13,\;0.06\right)$$

(29) $$\begin{array}{l} \mathrm{Model}\;3:AS=0.16_{IA}+0.20_{SH}+0.23_{SM}\\ +\;5.09\;\left(0.17,\;0.05\right) \end{array}$$

(30) $$\begin{array}{l} \mathrm{Model}\;4:AS=0.10_{IA}+0.15_{SH}+0.18_{SM}+0.21_{SSC}\\ +\;4.86\;\left(0.21,\;0.03\right) \end{array}$$

(31) $$\mathrm{Model}\;1:PS=0.26_{IA}+3.09\;\left(0.07\right)$$

(32) $$\mathrm{Model}\;2:PS=0.22_{IA}+0.25_{SH}+3.00\;\left(0.13,\;0.06\right)$$

(33) $$\begin{array}{l} \mathrm{Model}\;3:PS=0.16_{IA}+0.20_{SH}+0.23_{SM}\\ +\;2.92\;\left(0.17,\;0.05\right) \end{array}$$

(34) $$\begin{array}{l} \mathrm{Model}\;4:PS=0.10_{IA}+0.16_{SH}+0.17_{SM}+0.21_{SSC}\\ +\;2.87\;\left(0.20,\;0.03\right) \end{array}$$

**Table 4:** Prediction of specific students’ inputs to learning outcomes generally and in terms of CAPs.

Variable	Model	Predictors	β	*t*	*SE*
Learning outcomes generally	1	Innate ability	0.20^***^	6.00	2.19
	2	Innate ability	0.17^***^	5.05	2.18
		Students’ health	0.20^***^	6.07	1.71
	3	Innate ability	0.12^***^	3.43	2.23
		Students’ health	0.16^***^	4.96	1.72
		students’ motivation	0.19^***^	5.51	1.69
	4	Innate ability	0.07	1.93	2.30
		Students’ health	0.13^***^	3.75	1.75
		students’ motivation	0.14^***^	3.98	1.74
		Students’ social capital	0.18^***^	4.82	1.76
Cognitive skills	1	Innate ability	0.20^***^	5.91	2.11
	2	Innate ability	0.16^***^	4.96	2.09
		Students’ health	0.20^***^	6.00	1.64
	3	Innate ability	0.11^***^	3.36	2.14
		Students’ health	0.16^***^	4.90	1.65
		students’ motivation	0.19^***^	5.44	1.63
	4	Innate ability	0.07	1.88	2.21
		Students’ health	0.13^***^	3.70	1.68
		students’ motivation	0.14^***^	3.93	1.68
		Students’ social capital	0.18^***^	4.76	1.69
Affective skills	1	Innate ability	0.26^***^	8.08	0.06
	2	Innate ability	0.23^***^	6.99	0.06
		Students’ health	0.24^***^	7.45	0.04
	3	Innate ability	0.16^***^	4.98	0.06
		Students’ health	0.20^***^	6.11	0.04
		students’ motivation	0.23^***^	7.05	0.04
	4	Innate ability	0.10^***^	3.13	0.06
		Students’ health	0.15^***^	4.65	0.04
		students’ motivation	0.18^***^	5.19	0.04
		Students’ social capital	0.21^***^	5.99	0.05
Psychomotor skills	1	Innate ability	0.26^***^	7.90	0.03
	2	Innate ability	0.22^***^	6.79	0.03
		Students’ health	0.25^***^	7.59	0.03
	3	Innate ability	0.16^***^	4.84	0.03
		Students’ health	0.20^***^	6.28	0.03
		Students’ motivation	0.23^***^	6.84	0.03
	4	Innate ability	0.10^***^	3.02	0.03
		Students’ health	0.16^***^	4.85	0.03
		students’ motivation	0.17^***^	5.01	0.03
		Students’ social capital	0.21^***^	5.85	0.03

*** *p* < 0.001.

Where LO = Learning outcomes; CS = Cognitive skills; AS = Affective skills; PS = Psychomotor skills; IA = Innate ability; SH = Students health; SM = Students motivation; SSC = Students social capital.

## Discussion

This study was designed to examine the degree to which four students’ input (innate ability, health, motivation, and social capital) predict their learning outcomes across the cognitive, affective and psychomotor domains. A quantitative cross-sectional survey design was adopted for the study. This study discovered that innate ability, health, motivation and social capital combined predict students’ learning outcomes generally and in the dimensions of cognitive, affective and psychomotor domains. This finding is consistent with the result of Hindal et al. (2013a) that learners’ characteristics influence academic achievement significantly. In the same direction, other scholars (Apple et al., 2016; Espinosa, 2017) have also found that students’ characteristics predict their academic outcomes. Although these studies did not assess the composite prediction of students’ characteristics, the current study has contributed to the literature. Nevertheless, the similarity in the results of the studies shows a general agreement across different contexts. The result is not surprising because students’ learning ability is a product of their nature and nurture (environment). Therefore, it is no surprise that students who jointly possess nature-and environment-induced attributes such as innate ability, health, motivation and social capital to varying degrees can learn and achieve differently in secondary schools.

Relatively, this study showed that students’ innate ability predicts their learning outcomes generally and in the cognitive, affective and psychomotor domains. This study’s result provides further empirical support to other studies that earlier found that students’ innate ability determines the success of students in schools (Leslie et al., 2015; Meyer et al., 2015; Ito and McPherson, 2018; Heydera et al., 2020). Although the cited studies all focused on students’ cognitive domain while measuring achievement, the current study has addressed the shortcomings by proving that students’ innate ability is also a significant predictor of the affective and psychomotor domains. The result suggests that students with a solid innate ability tended to achieve better learning outcomes generally and in the cognitive, affective and psychomotor domains than those with a weak innate ability. The result of the current study might be attributed to students’ genetic differences, which empowers students differently. For instance, some students are high achievers because they originate from families with good intellectual genetic traits. Furthermore, because the genes in learners make them grasp course contents differently and at a different pace, those with a good grasp are likelier to achieve better than those on the opposite end.

Similarly, this study showed that students’ health has a significant individual prediction of their learning outcomes generally and in the cognitive, affective and psychomotor dimensions. The result corroborates another study that documented that school failure, grade repetition, and dropout are more likely for ill pupils than those who are otherwise healthy (Shaw et al., 2015). Therefore, nutritional deficiencies in the brain and body impact a child’s dietary condition (McIsaac et al., 2015). By implication, students with good health tended to demonstrate better learning outcomes (across the three domains) than those with poor health. An explanation for this result is that students cannot learn effectively when they are sick, hungry, depressed or addicted to substance use. Therefore, students’ poor learning outcomes among those with poor health conditions are attributable to the inability to process information, physical disability, lack of concentration, coordination and psychological imbalance. New studies also show that secondary school children who do better academically also engage in more healthy habits on an individual and population level (Grøtan et al., 2019; Luo et al., 2022). Furthermore, individuals who engage in risky activities, including not getting enough exercise, eating poorly, smoking, or abusing alcohol or other drugs, are more likely to do poorly in school and have a reduced chance of graduating (Bożek et al., 2020; López-Bueno et al., 2020; Akah et al., 2022; Owan et al., 2022).

Regarding motivation, this study discovered its significant relative prediction of students’ learning outcomes broadly and across the cognitive, affective and psychomotor dimensions. This result implies that the more students are motivated to learn, the higher their chances of maintaining acceptable learning outcomes in secondary schools. The result of this study strengthens the finding of researchers that also documented that students’ motivation relates positively to their academic achievement (Momany et al., 2015) and learning outcomes (Georgiou and Kyza, 2018; Won et al., 2020; Cho et al., 2021; Howard et al., 2021). Regarding the strength of the relationship, the current study found a weak predictive relationship between motivation and students’ learning outcomes generally and across the three domains. This result supports previous studies that found a weak positive correlation between motivation and students’ academic achievement (Dewangga and Nasaruddin, 2020; Rahardjo and Pertiwi, 2020). The result, however, differs slightly from other studies that found a modest link between motivation and students’ learning outcomes (Khin and Swe, 2018; Rosmayanti and Yanuarti, 2018; Ndruru et al., 2022). Despite the tiny difference, one major similarity between all the cited studies and the current one is that the relationship was proven to be statistically significant, although previous research has been mainly on the cognitive domain.

Furthermore, it was proven in the current study that students’ social capital is a significant predictor of their learning outcomes generally and across the three dimensions. The result implies that increments in students’ social capital is associated with increased student learning outcomes holistically and in the cognitive, affective and psychomotor dimensions. The result aligns with other studies that have identified social relationships as a determinant of students’ learning outcomes (Cho et al., 2007; Gablinske, 2014; Espinosa, 2017). The result is not surprising because a school is a social entity that requires a social network of relationships, and students may spend time and money building social networks in the hopes of receiving both personal and intellectual support in pursuit of their academic objectives (Cho et al., 2007; Gablinske, 2014). However, one major surprise in the results of the present study is that students’ social capital weakened the prediction of their innate ability for their overall and cognitive learning outcomes. The result suggests that students’ social ties with their colleagues could shift the onus of academic success from relying on one’s innate ability alone to social support from friends. By implication, a person can learn from colleagues and derive support from them to excel academically without relying on their abilities.

### Limitations and suggestions for further research

This study’s core strength is its use of a robust statistical procedure to link four predictors to three underresearched criterion variables. However, even though the study demonstrated strengths in its use of quantitative methods for results generalization to the broader population, the use of the method was also a critical drawback. While the quantitative technique allows generalizations to be made based on data from a substantial sample, it does not offer the opportunity to give a thorough justification for the relationships between the predictors and criterion variables in this research. Even though this in no way casts doubt on the findings of our study, future research should consider using a mixed methods approach to address the overlapping limitations of quantitative and qualitative approaches with their strengths. This study’s scope, which did not permit the inclusion of control or moderating factors such as students’ age, gender, class, and family background characteristics, among others, is another drawback. As a result, we could not determine, for example, whether the four inputs affect learning outcomes across the three domains equally for male and female students or younger and older students. However, it is commonly accepted that no research can provide a comprehensive response to all the issues raised by a topic at once. In addition, despite the best efforts of the researchers, no one study can fully account for all the control factors that potentially influence the link between two variables. We, therefore, suggest that future research use a multigroup analysis to investigate the impact of moderating factors in the connection between the four inputs to students’ learning outcomes across the three domains.

## Conclusion

This study provided evidence that students’ innate ability, health, motivation and social capital jointly predict their overall, cognitive, affective and psychomotor learning outcomes in secondary schools. The study also showed that these predictors are individually crucial in explaining the variance in students’ overall, cognitive, affective and psychomotor learning outcomes. The study proved that having sound health, positive motivation, high intellectual ability and social capital are crucial to boosting students’ overall performance across all subjects and promoting cognitive, affective and psychomotor outcomes. The study also proved that students seeking academic success could rely on their social capital without over-burdening their innate ability. This study contributes to the educational psychology literature by bridging the knowledge gap on the predictive relationship of students’ innate ability, health, motivation and social capital to their overall, affective and psychomotor learning outcomes. Although most previous studies have focused on students’ cognitive learning outcomes as predicted by innate ability, health, motivation, and social capital, the present study adds to it by testing their composite prediction.

## Implications and recommendations

The result of this study can be helpful in school management to provide services aimed at improving the school climate for students’ motivation and social capital. The result can also provide policymakers with a proper understanding of the constituents of learning outcomes and how policies can be aligned to secure quality student inputs for maximum education productivity. The study can help educational stakeholders (such as parents, teachers, school leaders, and the government) identify their roles in the education production process and how these roles can be effectively coordinated through a policy framework to aid the effective delivery of educational services in society. Lastly, curriculum planners are primarily concerned with cognitive outcomes of the educational process in schools, while other aspects of educational outcomes are sometimes not adequately captured in the schools’ planned contents, experiences and evaluation schedules in the Nigerian context. This study has, therefore, provided the need for the full articulation of non-cognitive (affective) and practical skills (psychomotor) measures into the school curriculum for better assessment of students’ holistic learning outcomes in schools. Based on the findings of this study, it is recommended that:

1. The government provides inclusive primary healthcare to enable students to access them when needed. This would keep both teachers and students healthy for quality teaching and learning.
2. Students should always be encouraged to maintain a positive perception of themselves and their abilities. This is critical in boosting their self-esteem and motivating them to learn for optimal learning outcomes.
3. Parents should ensure that students are given the right home environment to learn effectively. The food quality provided to learners at home should be nutritious to keep them healthy, sound and sharp for academic matters.
4. Students should often engage other colleagues and teachers in academic discussions, ask questions where there is a need for clarity and assist others that are weaker in learning. This will boost students’ social capital, making learning fun through collaboration and friendly support.

## Data availability statement

The raw data supporting the conclusions of this article will be made available by the authors, without undue reservation.

## Ethics statement

Ethical review and approval was not required for the study on human participants in accordance with the local legislation and institutional requirements. The patients/participants provided their written informed consent to participate in this study.

## Author contributions

VO contributed to the conceptualization, design, data collection, analysis, manuscript preparation and drafting process, editing and approval. JE contributed to the conceptualization, design, data collection, analysis, manuscript preparation and drafting process and approval. OC contributed to the data collection, financial support, revision and supervision. MA contributed to the design, data collection, technical support and approval. JO contributed to the data collection, manuscript revision, software and approval. MO contributed to the data collection, data cleaning, manuscript revision and approval. SO contributed to the data collection, manuscript revision and approval.

## Conflict of interest

The authors declare that the research was conducted in the absence of any commercial or financial relationships that could be construed as a potential conflict of interest.

## Publisher’s note

All claims expressed in this article are solely those of the authors and do not necessarily represent those of their affiliated organizations, or those of the publisher, the editors and the reviewers. Any product that may be evaluated in this article, or claim that may be made by its manufacturer, is not guaranteed or endorsed by the publisher.
